# Association between body mass index-for-age and slipped capital femoral epiphysis: the long-term risk for subsequent slip in patients followed until physeal closure

**DOI:** 10.1007/s11832-016-0731-y

**Published:** 2016-04-19

**Authors:** Michael W. Aversano, Payam Moazzaz, Anthony A. Scaduto, Norman Y. Otsuka

**Affiliations:** Department of Orthopedic Surgery, NYU Langone Medical Center Hospital for Joint Diseases, 301 East 17th Street, New York, NY USA; Department of Orthopaedic Surgery, David Geffen School of Medicine at University of California-Los Angeles, Los Angeles, CA USA; Orthopaedic Institute for Children/UCLA Department of Orthopaedic Surgery, Los Angeles, CA USA; Department of Pediatric Orthopaedics, The Children’s Hospital at Montefiore, Bronx, NY USA

**Keywords:** Slipped capital femoral epiphysis, SCFE, Body mass index, BMI, Physeal closure, Pediatric orthopaedic

## Abstract

**Background:**

Children who present with idiopathic slipped capital femoral epiphysis (SCFE) have an increased risk of developing bilateral disease. Predicting which patients will develop problems with bilateral hips is important for determining treatment algorithms. This is a retrospective observational study that evaluates the relationship and risk between body mass index (BMI)-for-age and unilateral and bilateral SCFE in patients followed until physeal closure.

**Methods:**

This is a retrospective study of all patients with SCFE presenting to one institution from 1998–2005. Using the Center for Disease Control (CDC) references, BMI-for-age was calculated for each patient. The patients were followed up until complete closure of the bilateral proximal femoral physes, which was considered completion of the study. Statistical analysis for significant differences between groups was performed using the Kruskal–Wallis test for equality of populations. A logistic regression, controlling for age and gender, was used to identify BMI-for-age as a risk factor and to determine the significance of the odds ratios (ORs) for the relevant categorical variables—obese, overweight and healthy weight.

**Results:**

Eighty patients (56 male, 24 female) presented to a single institution between 1998 and 2005 with a diagnosis of SCFE. The mean age of patients was 12.2 years at initial presentation (range 8.5–16). Forty-eight patients (32 male, 16 female) presented with unilateral SCFE, with 22 of the 48 patients having a BMI for-age percentile ≥95 %. Thirty-two patients (24 male, 8 female) presented with bilateral SCFE, with 29 of the 32 patients having a BMI-for-age percentile ≥95 %. Patients with a BMI-for-age ≥95 % had a significantly increased risk of presentation with bilateral slips (OR 4.83; relative risk [RR] 3.01; *p* < 0.05]. All but one patient in this study with bilateral SCFE or unilateral SCFE with subsequent contralateral involvement had a BMI-for-age ≥85 % (44 out of 45 patients). Additionally, the overall risk of developing bilateral SCFE until physeal closure with a BMI-for-age ≥95 % was significantly increased (OR 3.84; RR 2.02; *p* < 0.05; number needed to treat [NNT] 3.01).

**Conclusions:**

Previous work has established a relationship between BMI and SCFE. The CDC BMI-for-age growth charts more accurately measure obesity in the pediatric population compared to BMI and are therefore a more appropriate reference tool. This study demonstrates an association between obesity measured by BMI-for-age percentiles and SCFE. This study also demonstrates an association between BMI-for-age and risk for bilateral SCFE at presentation as well as overall incidence of developing bilateral SCFE in the obese pediatric population. By defining the at-risk population through BMI-for-age, physicians can screen the pediatric patient population and provide early strategies for therapeutic weight loss which may reduce the incidence of SCFE.

## Introduction

Slipped capital femoral epiphysis (SCFE) is the most common adolescent hip disorder, with an estimated prevalence of 2.13–10.8 per 100,000 in the United States [1, 2]. While most cases of SCFE are idiopathic, the fundamental etiology can be attributed to a combination of mechanical, endocrine and genetic components. Overwhelming shear stress across the physis ultimately results in biomechanical failure characterized by anterior-cranial-lateral movement of the proximal femoral metaphysis relative to the epiphysis [2–4].

Although SCFE may occur in children of healthy weight, the disorder is commonly associated with obesity [1–5]. Cadaver studies have suggested that increased forces may lead to SCFE in these obese children [6]. It has also been hypothesized that SCFE may be due to a failure of the structural integrity of the physis secondary to a genetic or acquired problem, such as that associated with endocrine disorders [7–12].

The preferred treatment for SCFE is generally surgical stabilization of the affected hip. In addition, at many institutions surgeons may choose to perform prophylactic pinning of the asymptomatic contralateral side [13–15]. As with any surgical procedure, internal fixation of SCFE is not without risk and morbidity including possible avascular necrosis and chondrolysis [16, 17]. As a result, prophylactic pinning was traditionally performed only in high-risk patients such as those with younger bone age at presentation, renal failure, or endocrinopathies [18–22]. As more epidemiologic data become available, a number of studies have suggested that prophylactic pinning may be safer and preferable to observation, citing a high prevalence of long-term osteoarthric sequela with missed SCFE [14, 15, 23–26]. Nevertheless, controversy stills exists regarding the advisability of prophylactic pinning of the contralateral hip.

Body mass index (BMI) has also been suggested as a risk factor for SCFE [5, 27, 28]. It has been consistently demonstrated that patients presenting with SCFE have a higher than average BMI, and that children with bilateral SCFE have a higher BMI than children with unilateral SCFE [1, 2, 6, 29–31]. Patients who present with unilateral SCFE and progress to bilateral disease have also been previously found to have a significantly greater BMI than patients with unilateral SCFE who did not progress [28].

**Fig. 1:**
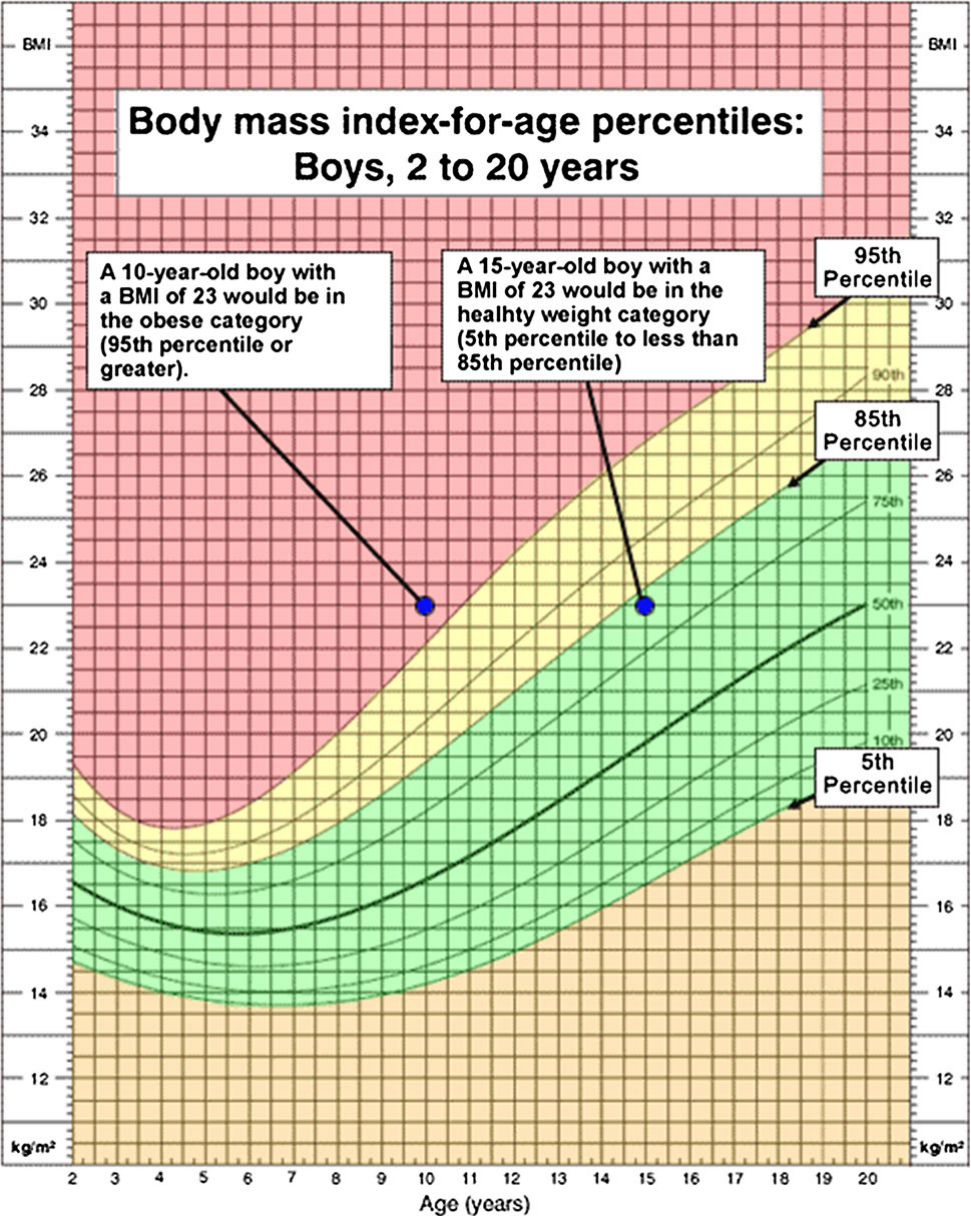
Body mass index-for-age percentiles [31]

The recently developed BMI-for-age percentile has been shown to more effectively evaluate obesity in the pediatric population [29]. Although BMI is calculated in the same way for children and adults, the criteria used to interpret the meaning of BMI in children is different than adults as the amount of body fat changes with age [30] (Fig. 1). For example, a 10-year-old boy with a BMI of 23 would be considered obese (≥95th percentile) while a 15-year-old boy with a BMI of 23 would be considered healthy (5th–85th percentile) [31]. Methods for evaluating obesity also depend on regional population disparities as demonstrated by differences in BMI calculations between data collection references such as Must, Dallal and Dietz, Cole et al., and Kuczmarski et al. [32–35]. Additionally, studies such as those by Loder and colleagues demonstrate that ethnicity differences must be considered when evaluating certain patient populations [36, 37].

The purpose of this study was to examine the relationship between BMI-for-age and unilateral versus bilateral presentation of SCFE as well to clinically follow these patients until radiographic physeal closure to ascertain their risk for future contralateral slip.

## Materials and methods

This was a retrospective study of all patients with SCFE presenting to one institution from 1998–2005. All charts and radiographs were available for review. Height and weight at presentation were used to calculate BMI using the formula BMI = weight (kg)/height^2^ (m^2^). The Center for Disease Control (CDC) 2000 BMI-for-age growth charts were used to calculate individual BMI-for-age percentiles. According to the CDC growth charts, children with BMI-for-age levels ≥95th percentile are considered obese and those between the 85th and 95th percentiles are considered overweight [29]. Patients were divided into one of three groups—unilateral presentation, bilateral presentation, or unilateral presentation with subsequent contralateral slip. BMI-for-age percentile was calculated for each presentation.

The presence of SCFE was determined radiographically. All radiographs were evaluated by both a board-certified pediatric orthopedic surgeon as well as a board-certified radiologist. In correlation with the physical examination and history, criteria for diagnosis of slips were based on (1) widening and irregularity of the physis, (2) evaluation of Klein’s line or a Trethowan sign, and (3) discrepancy between the head-shaft angle of the hips as seen on anteroposterior and frog-leg lateral views of the pelvis. The patients were followed up until complete closure of the bilateral proximal femoral physes, which was considered completion of the study. Statistical analysis for significant differences between groups was performed using the Kruskal–Wallis test for equality of populations. The level of significance was set at 5 %. A logistic regression, controlling for age and gender, was used to identify BMI-for-age as a risk factor and to determine the significance of the odds ratios (ORs) for the relevant categorical variables—obese, overweight and healthy weight.

## Results

Eighty patients (56 male, 24 female) presented to a single institution between 1998 and 2005 with a diagnosis of SCFE. The mean age of patients was 12.2 years at initial presentation (range 8.5–16). The males were significantly older with a mean age of 13.1 years (range 8.5–16) compared to a mean age of 11.7 years (range 9–14) for females (*p* < 0.01). None of the children showed evidence of endocrinopathy or renal failure. The mean duration of follow-up was 2.2 years.

**Fig. 2:**
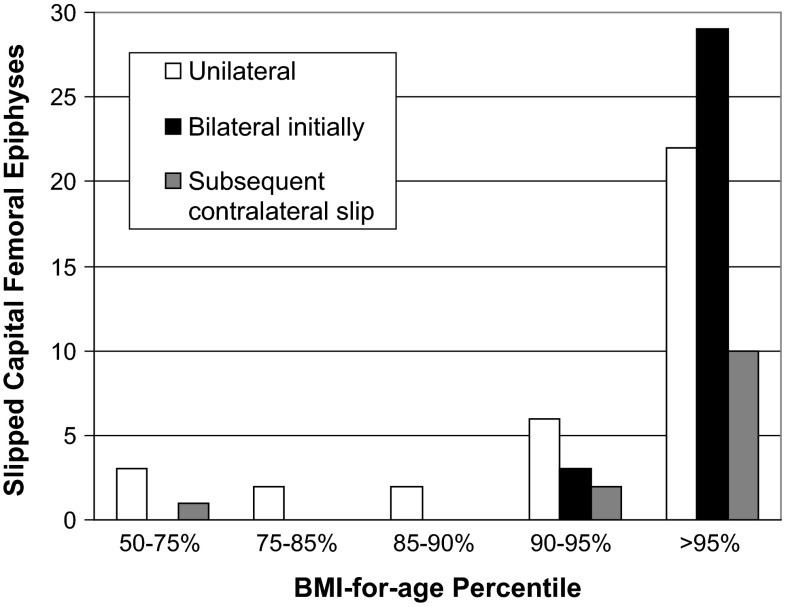
Unilateral, bilateral, and subsequent SCFE by BMI-for-age percentile

The mean BMI for all patients included in the study was 29.7 kg/m^2^ (range 19.0–47.5 kg/m^2^). Forty-eight patients (32 male, 16 female) presented with unilateral SCFE, with 22 of the 48 patients having a BMI-for-age percentile ≥95 %. Thirty-two patients (24 male, 8 female) presented with bilateral SCFE, with 29 of the 32 patients having a BMI-for-age percentile ≥95 %. Thirteen patients (7 male, 6 female) developed a subsequent contralateral slip, with 10 of the 13 patients having a BMI-for-age percentile ≥95 % (Fig. 2).

Patients with a BMI-for-age ≥95 % represented 63 % of patients with unilateral SCFE (22/35), 91 % of patients with bilateral SCFE (29/32), and 77 % of patients who developed a contralateral slip (10/13). Patients with a BMI-for-age between 85 and 95 % represented 23 % of patients with unilateral SCFE (8/35), 9 % of patients with bilateral SCFE (3/32), and 15 % of patients who developed a contralateral slip (2/13) (Table 1).

**Table 1:** Distribution of patients based on BMI-for-age and diagnosis of SCFE

BMI-for-age	Unilateral	Unilateral+^a^	Bilateral
<85 %	5	1	0
85–95 %	8	2	3
>95 %	22	10	29
Totals	35	13	32

^a^ *Unilateral*+ represents the group of patients that initially presented with unilateral SCFE and subsequently developed SCFE in the contralateral hip

Sixty-three percent of patients with a BMI-for-age ≥95 % presented with a bilateral SCFE or presented with unilateral SCFE and developed a subsequent contralateral SCFE. All but one patient in this study with bilateral SCFE or unilateral SCFE with subsequent contralateral involvement had a BMI-for-age ≥85 % (44 out of 45 patients).

Patients with a BMI-for-age ≥95 % had a significantly increased risk of presentation with bilateral slips (OR 4.83; RR 3.01; *p* < 0.05]. There was no significant difference in the development of future contralateral slip (OR 1.97; RR 1.67; *p* = 0.38) when isolating patients who presented with unilateral SCFE and went on to develop bilateral pathology. The overall risk of developing bilateral SCFE until physeal closure with a BMI-for-age ≥95 %, however, was significantly increased (OR 3.84; RR 2.02; *p* < 0.05). The number to be treated (NNT) was 3.01, suggesting that pinning all hips in patients with a BMI-for-age >95 % would mean pinning three hips in order to prevent one hip from progression to bilateral slips. Patients with a BMI-for-age between 85 and 95 % did not have a significantly increased risk of presentation with bilateral slips (OR 4.33; RR 3.50; *p* = 0.36) or development of future contralateral slip (OR 1.25; RR 1.20; NNT 30; *p* = 0.87) compared to individuals with a BMI-for-age < 85 %.

## Discussion and conclusion

Although the exact etiology of SCFE remains unproven, many previous investigators have suggested an association with obesity and possible increased shear stress across the physis leading to an increased incidence of SCFE in this population [6, 7, 10]. The results of this study support this hypothesis as the majority of patients in this study were classified as overweight or obese. Previous work has established a relationship between BMI and SCFE [1, 27, 28, 38]. This study uses the CDC BMI-for-age growth charts to more accurately measure obesity in the pediatric population compared to BMI. However, the CDC reference is just one way of calculating BMI-for-age; although it covers a large sampling of representative populations, it is not all-inclusive. The CDC references for the United States were based on data collected from European Americans, African Americans and Mexican Americans sampled between 1988 and 1994 in the NHANES III study. When compared to other models it demonstartes an increased proportion of the population in the overweight and obesity categories [35, 39].

This study demonstrates an association between obesity as measured by BMI-for-age percentiles and SCFE. This study also elucidates an association between BMI-for-age and risk for bilateral SCFE both at initial presentation and throughout the remaining growth period. While there was no significant association between BMI-for-age and subsequent contralateral slip after initial presentation, our study was likely underpowered to prove this given the smaller sub-group size. If the assumption was made that all patients with bilateral SCFE at initial presentation at some point in their clinical history had unilateral SCFE and then developed bilateral pathology, the adjusted calculations would then indicate a statistically signficant correlation between BMI and SCFE.

In addition to larger long-term prospective studies, further work is needed to establish the effect of weight reduction on the incidence of future contralateral slips in this population. As suggested by Loder et al., physiologic or bone age is less variable than the chronologic age when discussing risk for SCFE and this may be one of the limitations of our study [40]. Furthermore, as this is an observational study continuous variables, such as age at onset and relation to peak height velocity, are possible cofounding factors during OR analysis.

The prevalence of bilateral involvement in this study is also consistent with previously published reports of an 18–63 % prevalence of bilateral disease, with 40 % (32/80) of patients presenting with bilateral SCFE in this study [23, 37, 41]. This study also demonstrates that patients with bilateral SCFE had a significantly higher BMI-for-age than children with unilateral SCFE. Patients who presented with unilateral SCFE who progressed to bilateral disease also had a significantly higher BMI-for-age than patients with unilateral disease who did not progress.

Patients with a BMI-for-age ≥95 % are at a significantly increased risk of developing SCFE. If clinically correlated, obese patients warrant a high index of suspicion for bilateral disease given their increased presentation with bilateral SCFE and propensity for future development of a contralateral slip. This study suggests that strong consideration should be given to prophylactic stabilization of the contralateral hip in patients with SCFE presenting with a BMI-for-age ≥95 %. In addition, by defining the at-risk population through BMI-for-age, physicians can screen the pediatric patient population and provide early strategies for therapeutic weight loss which may reduce the incidence of SCFE.
